# Crystal structure of *catena*-poly[silver(I)-μ-l-tyrosinato-κ^2^ *O*:*N*]

**DOI:** 10.1107/S2056989015001905

**Published:** 2015-02-04

**Authors:** Aqsa Yousaf, Muhammad Nawaz Tahir, Abdul Rauf, Shafique Ahmad Awan, Saeed Ahmad

**Affiliations:** a Department of Chemistry, University of Engineering and Technology, Lahore 54890, Pakistan; b Department of Physics, University of Sargodha, Sargodha, Punjab, Pakistan; c PAEC, PO Box No. 1114, Islamabad GPO 44000, Pakistan

**Keywords:** crystal structure, one-dimensional silver(I) coordination polymer, l-tyrosinate, hydrogen bonding

## Abstract

The title compound, [Ag(C_9_H_10_NO_3_)]_*n*_, is a polymeric silver(I) complex of l-tyrosine. The Ag^I^ atom is connected to N and O atoms of two different l-tyrosine ligands in an almost linear arrangement, with an N^i^—Ag—O1 bond angle of 173.4 (2)° [symmetry code: (i) *x* + 1, *y*, *z*]. The Ag—N^i^ and Ag—O bond lengths are 2.156 (5) and 2.162 (4) Å, respectively. The polymeric chains extend along the crystallographic *a* axis. Strong hydrogen bonds of the N—H⋯O and O—H⋯O types and additional C—H⋯O inter­actions connect these chains into a double-layer polymeric network in the *ab* plane.

## Related literature   

For related structures and studies, see: Ahmad *et al.* (2006 ▸); Kasuga *et al.* (2011 ▸); Nomiya *et al.* (2000 ▸); Nomiya & Yokoyama (2002 ▸).
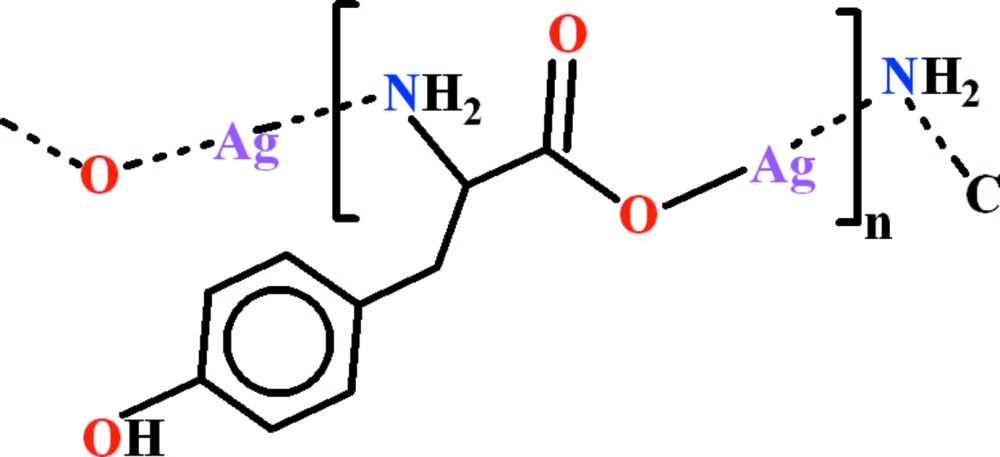


## Experimental   

### Crystal data  

[Ag(C_9_H_10_NO_3_)]
*M* *_r_* = 288.05
Monoclinic, 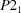

*a* = 7.2944 (5) Å
*b* = 7.1464 (5) Å
*c* = 9.2736 (7) Å
β = 101.546 (4)°
*V* = 473.64 (6) Å^3^
*Z* = 2
Mo *K*α radiation
μ = 2.11 mm^−1^
*T* = 296 K
0.34 × 0.20 × 0.18 mm

### Data collection  

Bruker Kappa APEXII CCD diffractometer
Absorption correction: multi-scan (*SADABS*; Bruker, 2005 ▸) *T*_min_ = 0.538, *T*_max_ = 0.701
4086 measured reflections
1816 independent reflections
1752 reflections with *I* > 2σ(*I*)
*R*_int_ = 0.023

### Refinement  

*R*[*F* ^2^ > 2σ(*F* ^2^)] = 0.022
*wR*(*F* ^2^) = 0.052
*S* = 1.11
1816 reflections
134 parameters
1 restraint
H atoms treated by a mixture of independent and constrained refinement
Δρ_max_ = 0.28 e Å^−3^
Δρ_min_ = −0.48 e Å^−3^
Absolute structure: Flack *x* determined using 751 quotients [(*I* ^+^)−(*I* ^−^)]/[(*I* ^+^)+(*I* ^−^)] (Parsons *et al.*, 2013 ▸)
Absolute structure parameter: 0.04 (2)

### 

Data collection: *APEX2* (Bruker, 2007 ▸); cell refinement: *SAINT* (Bruker, 2007 ▸); data reduction: *SAINT*; program(s) used to solve structure: *SHELXS97* (Sheldrick, 2008 ▸); program(s) used to refine structure: *SHELXL97* (Sheldrick, 2008 ▸); molecular graphics: *ORTEP-3 for Windows* (Farrugia, 2012 ▸) and *PLATON* (Spek, 2009 ▸); software used to prepare material for publication: *WinGX* (Farrugia, 2012 ▸) and *PLATON*.

## Supplementary Material

Crystal structure: contains datablock(s) global, I. DOI: 10.1107/S2056989015001905/im2459sup1.cif

Structure factors: contains datablock(s) I. DOI: 10.1107/S2056989015001905/im2459Isup2.hkl

**Fig. 1.**
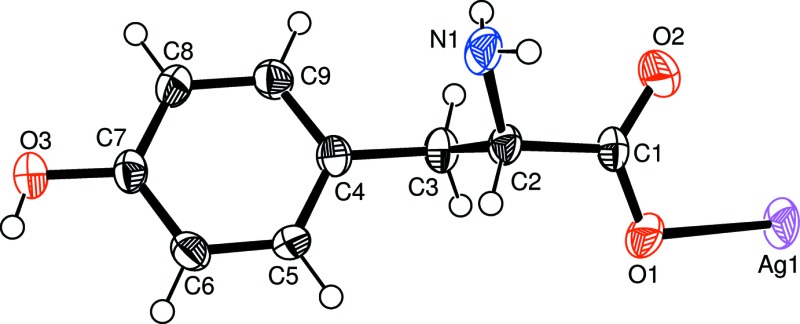
View of the asymmetric unit of the title compound. Thermal ellipsoids are drawn at the 50% probability level. H atoms are shown by small circles of arbitrary radii.

. DOI: 10.1107/S2056989015001905/im2459fig1.tif
View of the asymmetric unit of the title compound. Thermal ellipsoids are drawn at the 50% probability level. H atoms are shown by small circles of arbitrary radii.

**Fig. 2.**
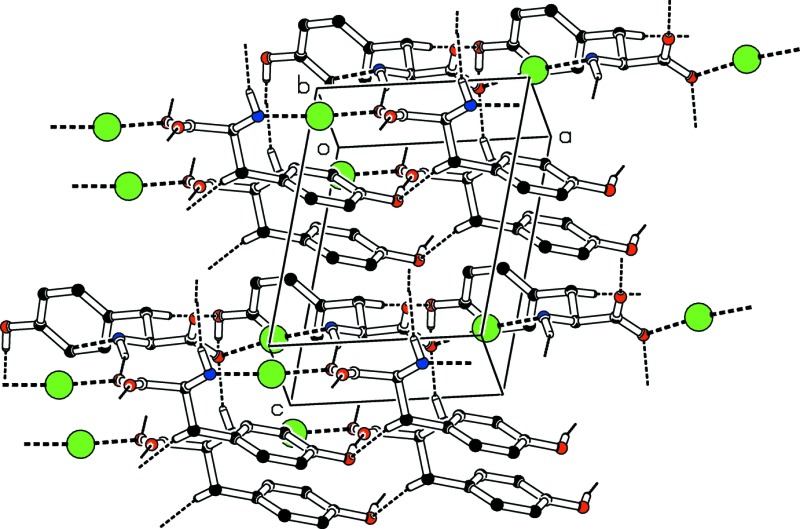
The partial packing (PLATON; Spek, 2009) showing the polymeric network due to C—H···O, N—H···O and O—H···O interactions. H atoms not involved in hydrogen-bonding interactions are omitted for clarity.

PLATON . DOI: 10.1107/S2056989015001905/im2459fig2.tif
The partial packing (*PLATON*; Spek, 2009) showing the polymeric network due to C—H⋯O, N—H⋯O and O—H⋯O inter­actions. H atoms not involved in hydrogen-bonding inter­actions are omitted for clarity.

CCDC reference: 1046166

Additional supporting information:  crystallographic information; 3D view; checkCIF report

## Figures and Tables

**Table 1:** Hydrogen-bond geometry (, )

*D*H*A*	*D*H	H*A*	*D* *A*	*D*H*A*
O3H3O2^i^	0.82	1.91	2.710(7)	166
N1H1*B*O1^ii^	0.87(7)	2.19(7)	2.988(6)	152(5)
C2H2O2^iii^	0.98	2.63	3.589(7)	168
C3H3*B*O3^iv^	0.97	2.48	3.441(7)	171

Symmetry codes: (i) 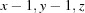; (ii) 
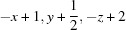
; (iii) 
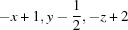
; (iv) 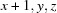.

## supplementary crystallographic information

### S1. Comment

Silver(I) complexes of amino acids are important from a medicinal point of view because of their effective biological activities against bacteria, yeasts and moulds (Ahmad *et al.* 2006; Kasuga *et al.* 2011; Nomiya *et al.* 2000; Nomiya & Yokoyama 2002). These complexes usually exist in the form of polymers and the Ag—O and Ag—N bonds play an important role in exhibiting a wide spectrum of antimicrobial activities. The Ag—O bonding complexes can readily undergo ligand replacement with sulfur containing biological ligands such as proteins (Ahmad *et al.* 2006; Kasuga *et al.* 2011; Nomiya *et al.* 2000; Nomiya & Yokoyama, 2002). The crystal structures of these complexes are also characterized by strong hydrogen bonding. The present report is concerned with the crystal structure of a new polymeric silver(I) complex of *L*-tyrosine (Fig. 1), which has been synthesized for various studies.

In (I) the part of acetate group A (C1/C2/O1/O2) is planar with r. m. s. deviation of 0.0065 Å. The attached N-atom is at a distance of 0.614 (9) Å from the plane A. The 4-methylphenol group B (C3—C9/O3) also attached at the same C-atom is planar with r. m. s. deviation of 0.0032 Å. The dihedral angle between A/B is 21.3 (3)°. Silver atoms are coordinted to *L*-tyrosine through the deprotonated O atom of the carboxyl group and the amino group of another amino acid residue. The Ag1–N1^i^ [i = *x* + 1, *y*, *z*] and Ag1–O1 bond distances are almost equal and have values of 2.156 (5) and 2.162 (4) Å, respectively. The N1^i^–Ag1–O1 bond angle is 173.4 (2)° due to which the silver atoms are at a separation of 7.2944 (7) Å in this polymeric complex. The polymeric chains are oriented along the crystallographic *a*-axis. Polymeric chains are linked to layers in the *ab* plane by O—H···O hydrogen bonds and two of theses layers are additionally linked to a double layer structure by N—H···O hydrogen bonds. This arrangement is further stabilized by weak C—H···O hydrogen bonds (Table 1, Fig. 2). Additionally, C—H···π interactions between benzene rings are observed. Due to these interactions molecules are arranged in the form of a two-dimensional polymeric network with base vectors [1 0 0], [0 1 0] in the (001) plane.

### S2. Experimental

*L*-Tyrosine (0.18 g, 1.0 mmol) was disolved in 10 ml water by adding 10 drops of 1.0 *M* NaOH. AgNO_3_ (0.17 g, 1.0 mmol) was disolved in 10 ml of acetonitrile. The *L*-tyrosine solution was slowly added to the AgNO_3_ solution and the resulting mixture after filtration was kept in the refrigerator at 0°C for crystallization. After three days, colorless needles of (I) were obtained (yield: 20%, m.p = 547 K).

### S3. Refinement

The coordinates of H-atoms of the NH_2_ group were obtained from the Fourier map and refined isotropically. The other H-atoms were positioned geometrically (C–H = 0.93–0.98 Å, O—H = 0.82 Å) and refined as riding with *U*_iso_(H) = *xU*_eq_(C, N, O) with *x* = 1.5 for hydroxy and *x* = 1.2 for other H-atoms.

### Crystal data

**Table:**

[Ag(C_9_H_10_NO_3_)]	*F*(000) = 284
*M**_r_* = 288.05	*D*_x_ = 2.020 Mg m^−^^3^
Monoclinic, *P*2_1_	Mo *K*α radiation, λ = 0.71073 Å
*a* = 7.2944 (5) Å	Cell parameters from 1753 reflections
*b* = 7.1464 (5) Å	θ = 2.9–26.0°
*c* = 9.2736 (7) Å	µ = 2.11 mm^−^^1^
β = 101.546 (4)°	*T* = 296 K
*V* = 473.64 (6) Å^3^	Block, colorless
*Z* = 2	0.34 × 0.20 × 0.18 mm

### Data collection

**Table:**

Bruker Kappa APEXII CCD diffractometer	1816 independent reflections
Radiation source: fine-focus sealed tube	1752 reflections with *I* > 2σ(*I*)
Graphite monochromator	*R*_int_ = 0.023
Detector resolution: 8.00 pixels mm^-1^	θ_max_ = 26.0°, θ_min_ = 2.9°
ω scans	*h* = −8→8
Absorption correction: multi-scan (*SADABS*; Bruker, 2005)	*k* = −8→8
*T*_min_ = 0.538, *T*_max_ = 0.701	*l* = −11→11
4086 measured reflections	

### Refinement

**Table:**

Refinement on *F*^2^	Secondary atom site location: difference Fourier map
Least-squares matrix: full	Hydrogen site location: inferred from neighbouring sites
*R*[*F*^2^ > 2σ(*F*^2^)] = 0.022	H atoms treated by a mixture of independent and constrained refinement
*wR*(*F*^2^) = 0.052	*w* = 1/[σ^2^(*F*_o_^2^) + (0.0222*P*)^2^ + 0.0305*P*] where *P* = (*F*_o_^2^ + 2*F*_c_^2^)/3
*S* = 1.11	(Δ/σ)_max_ = 0.001
1816 reflections	Δρ_max_ = 0.28 e Å^−^^3^
134 parameters	Δρ_min_ = −0.48 e Å^−^^3^
1 restraint	Absolute structure: Flack *x* determined using 751 quotients [(*I*^+^)-(*I*^-^)]/[(*I*^+^)+(*I*^-^)] (Parsons *et al.*, 2013)
Primary atom site location: structure-invariant direct methods	Absolute structure parameter: 0.04 (2)

### Special details

**Table:**

Geometry. All e.s.d.'s (except the e.s.d. in the dihedral angle between two l.s. planes) are estimated using the full covariance matrix. The cell e.s.d.'s are taken into account individually in the estimation of e.s.d.'s in distances, angles and torsion angles; correlations between e.s.d.'s in cell parameters are only used when they are defined by crystal symmetry. An approximate (isotropic) treatment of cell e.s.d.'s is used for estimating e.s.d.'s involving l.s. planes.
Refinement. Refinement of *F*^2^ against ALL reflections. The weighted *R*-factor *wR* and goodness of fit *S* are based on *F*^2^, conventional *R*-factors *R* are based on *F*, with *F* set to zero for negative *F*^2^. The threshold expression of *F*^2^ > σ(*F*^2^) is used only for calculating *R*-factors(gt) *etc*. and is not relevant to the choice of reflections for refinement. *R*-factors based on *F*^2^ are statistically about twice as large as those based on *F*, and *R*-factors based on ALL data will be even larger.

### Fractional atomic coordinates and isotropic or equivalent isotropic displacement parameters (Å^2^)

**Table:**

	*x*	*y*	*z*	*U*_iso_*/*U*_eq_	
Ag1	0.95099 (4)	0.57540 (7)	0.87242 (4)	0.04160 (14)	
O1	0.6862 (5)	0.4646 (5)	0.9045 (4)	0.0364 (8)	
O2	0.5870 (6)	0.7436 (5)	0.8171 (6)	0.0493 (10)	
O3	−0.4102 (5)	0.0526 (9)	0.6482 (4)	0.0418 (12)	
H3	−0.4074	−0.0285	0.7111	0.063*	
N1	0.2293 (6)	0.6694 (6)	0.8634 (6)	0.0367 (10)	
H1A	0.219 (9)	0.716 (8)	0.781 (7)	0.044*	
H1B	0.282 (9)	0.725 (9)	0.945 (7)	0.044*	
C1	0.5594 (5)	0.5852 (11)	0.8581 (4)	0.0270 (9)	
C2	0.3595 (7)	0.5138 (6)	0.8509 (5)	0.0265 (11)	
H2	0.3601	0.4267	0.9326	0.032*	
C3	0.2963 (7)	0.4091 (7)	0.7071 (5)	0.0307 (10)	
H3A	0.2921	0.4963	0.6264	0.037*	
H3B	0.3892	0.3146	0.6989	0.037*	
C4	0.1078 (7)	0.3152 (6)	0.6902 (5)	0.0273 (10)	
C5	0.0880 (7)	0.1543 (6)	0.7700 (5)	0.0312 (10)	
H5	0.1925	0.1052	0.8327	0.037*	
C6	−0.0825 (6)	0.0651 (10)	0.7588 (5)	0.0312 (9)	
H6	−0.0914	−0.0417	0.8143	0.037*	
C7	−0.2405 (6)	0.1346 (6)	0.6650 (5)	0.0290 (11)	
C8	−0.2230 (7)	0.2942 (7)	0.5838 (6)	0.0336 (11)	
H8	−0.3272	0.3422	0.5200	0.040*	
C9	−0.0515 (7)	0.3826 (7)	0.5972 (5)	0.0324 (11)	
H9	−0.0429	0.4900	0.5423	0.039*	

### Atomic displacement parameters (Å^2^)

**Table:**

	*U*^11^	*U*^22^	*U*^33^	*U*^12^	*U*^13^	*U*^23^
Ag1	0.01769 (18)	0.0458 (2)	0.0638 (3)	−0.0039 (2)	0.01412 (15)	−0.0078 (3)
O1	0.0186 (19)	0.044 (2)	0.047 (2)	0.0018 (16)	0.0080 (16)	0.0041 (17)
O2	0.034 (2)	0.038 (2)	0.077 (3)	−0.0093 (18)	0.015 (2)	0.0112 (19)
O3	0.0268 (16)	0.039 (3)	0.057 (2)	−0.009 (2)	0.0010 (14)	0.004 (2)
N1	0.020 (2)	0.039 (2)	0.052 (3)	−0.0001 (19)	0.010 (2)	−0.006 (2)
C1	0.0209 (19)	0.031 (2)	0.030 (2)	−0.006 (3)	0.0076 (15)	−0.007 (3)
C2	0.019 (3)	0.031 (3)	0.031 (3)	−0.0017 (16)	0.0073 (19)	0.0005 (16)
C3	0.025 (3)	0.037 (3)	0.032 (3)	−0.006 (2)	0.009 (2)	−0.003 (2)
C4	0.027 (2)	0.029 (2)	0.025 (2)	−0.0037 (19)	0.005 (2)	−0.0037 (18)
C5	0.027 (2)	0.027 (2)	0.036 (3)	0.0020 (19)	−0.002 (2)	0.0040 (19)
C6	0.035 (2)	0.024 (2)	0.034 (2)	−0.004 (3)	0.0055 (17)	0.000 (3)
C7	0.024 (2)	0.029 (3)	0.033 (3)	−0.0052 (17)	0.005 (2)	−0.0073 (17)
C8	0.025 (3)	0.038 (3)	0.035 (3)	−0.002 (2)	−0.001 (2)	0.001 (2)
C9	0.034 (3)	0.033 (2)	0.030 (3)	−0.003 (2)	0.004 (2)	0.0053 (19)

### Geometric parameters (Å, º)

**Table:**

Ag1—N1^i^	2.156 (4)	C3—C4	1.510 (6)
Ag1—O1	2.162 (4)	C3—H3A	0.9700
O1—C1	1.275 (7)	C3—H3B	0.9700
O2—C1	1.224 (9)	C4—C9	1.388 (7)
O3—C7	1.350 (6)	C4—C5	1.390 (6)
O3—H3	0.8200	C5—C6	1.383 (7)
N1—C2	1.482 (6)	C5—H5	0.9300
N1—Ag1^ii^	2.156 (4)	C6—C7	1.390 (7)
N1—H1A	0.83 (6)	C6—H6	0.9300
N1—H1B	0.87 (7)	C7—C8	1.386 (6)
C1—C2	1.534 (6)	C8—C9	1.385 (7)
C2—C3	1.518 (7)	C8—H8	0.9300
C2—H2	0.9800	C9—H9	0.9300
			
N1^i^—Ag1—O1	173.41 (18)	C4—C3—H3B	108.6
C1—O1—Ag1	108.3 (3)	C2—C3—H3B	108.6
C7—O3—H3	109.5	H3A—C3—H3B	107.5
C2—N1—Ag1^ii^	113.1 (3)	C9—C4—C5	117.0 (4)
C2—N1—H1A	100 (4)	C9—C4—C3	122.8 (4)
Ag1^ii^—N1—H1A	105 (4)	C5—C4—C3	120.1 (4)
C2—N1—H1B	103 (4)	C6—C5—C4	122.0 (5)
Ag1^ii^—N1—H1B	111 (4)	C6—C5—H5	119.0
H1A—N1—H1B	124 (6)	C4—C5—H5	119.0
O2—C1—O1	125.2 (4)	C5—C6—C7	120.2 (5)
O2—C1—C2	120.6 (5)	C5—C6—H6	119.9
O1—C1—C2	114.2 (6)	C7—C6—H6	119.9
N1—C2—C3	110.6 (4)	O3—C7—C8	118.5 (5)
N1—C2—C1	111.4 (5)	O3—C7—C6	122.9 (5)
C3—C2—C1	108.7 (4)	C8—C7—C6	118.6 (5)
N1—C2—H2	108.7	C9—C8—C7	120.4 (5)
C3—C2—H2	108.7	C9—C8—H8	119.8
C1—C2—H2	108.7	C7—C8—H8	119.8
C4—C3—C2	114.8 (4)	C8—C9—C4	121.8 (4)
C4—C3—H3A	108.6	C8—C9—H9	119.1
C2—C3—H3A	108.6	C4—C9—H9	119.1
			
Ag1—O1—C1—O2	−7.0 (6)	C2—C3—C4—C5	−73.4 (6)
Ag1—O1—C1—C2	170.8 (3)	C9—C4—C5—C6	−0.5 (7)
Ag1^ii^—N1—C2—C3	63.8 (5)	C3—C4—C5—C6	179.3 (5)
Ag1^ii^—N1—C2—C1	−175.2 (3)	C4—C5—C6—C7	0.6 (7)
O2—C1—C2—N1	−28.0 (7)	C5—C6—C7—O3	179.2 (5)
O1—C1—C2—N1	154.2 (4)	C5—C6—C7—C8	−0.1 (7)
O2—C1—C2—C3	94.2 (6)	O3—C7—C8—C9	−179.8 (5)
O1—C1—C2—C3	−83.6 (5)	C6—C7—C8—C9	−0.4 (7)
N1—C2—C3—C4	−62.6 (5)	C7—C8—C9—C4	0.5 (7)
C1—C2—C3—C4	174.7 (5)	C5—C4—C9—C8	0.0 (7)
C2—C3—C4—C9	106.5 (5)	C3—C4—C9—C8	−179.8 (4)

Symmetry codes: (i) *x*+1, *y*, *z*; (ii) *x*−1, *y*, *z*.

### Hydrogen-bond geometry (Å, º)

**Table:**

*D*—H···*A*	*D*—H	H···*A*	*D*···*A*	*D*—H···*A*
O3—H3···O2^iii^	0.82	1.91	2.710 (7)	166
N1—H1*B*···O1^iv^	0.87 (7)	2.19 (7)	2.988 (6)	152 (5)
C2—H2···O2^v^	0.98	2.63	3.589 (7)	168
C3—H3*B*···O3^i^	0.97	2.48	3.441 (7)	171

Symmetry codes: (i) *x*+1, *y*, *z*; (iii) *x*−1, *y*−1, *z*; (iv) −*x*+1, *y*+1/2, −*z*+2; (v) −*x*+1, *y*−1/2, −*z*+2.
