# Supplementary figures and images for: Crystal structure of catena-poly[silver(I)-μ-l-tyrosinato-κ2 O:N]
Source: Acta Crystallogr E Crystallogr Commun. 2015 Feb 4;71(Pt 3):m50–1. doi: 10.1107/S2056989015001905 (PMC4350688; doi:10.1107/S2056989015001905)

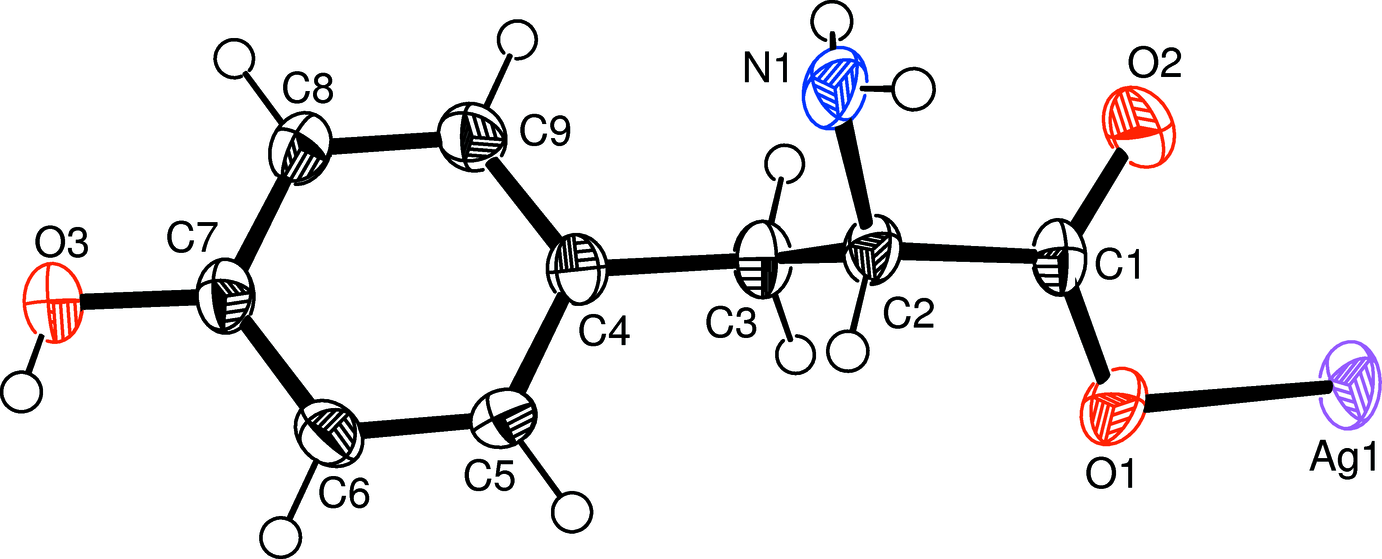

Supplement: Supplementary file 3 [file e-71-00m50-fig1.tif]

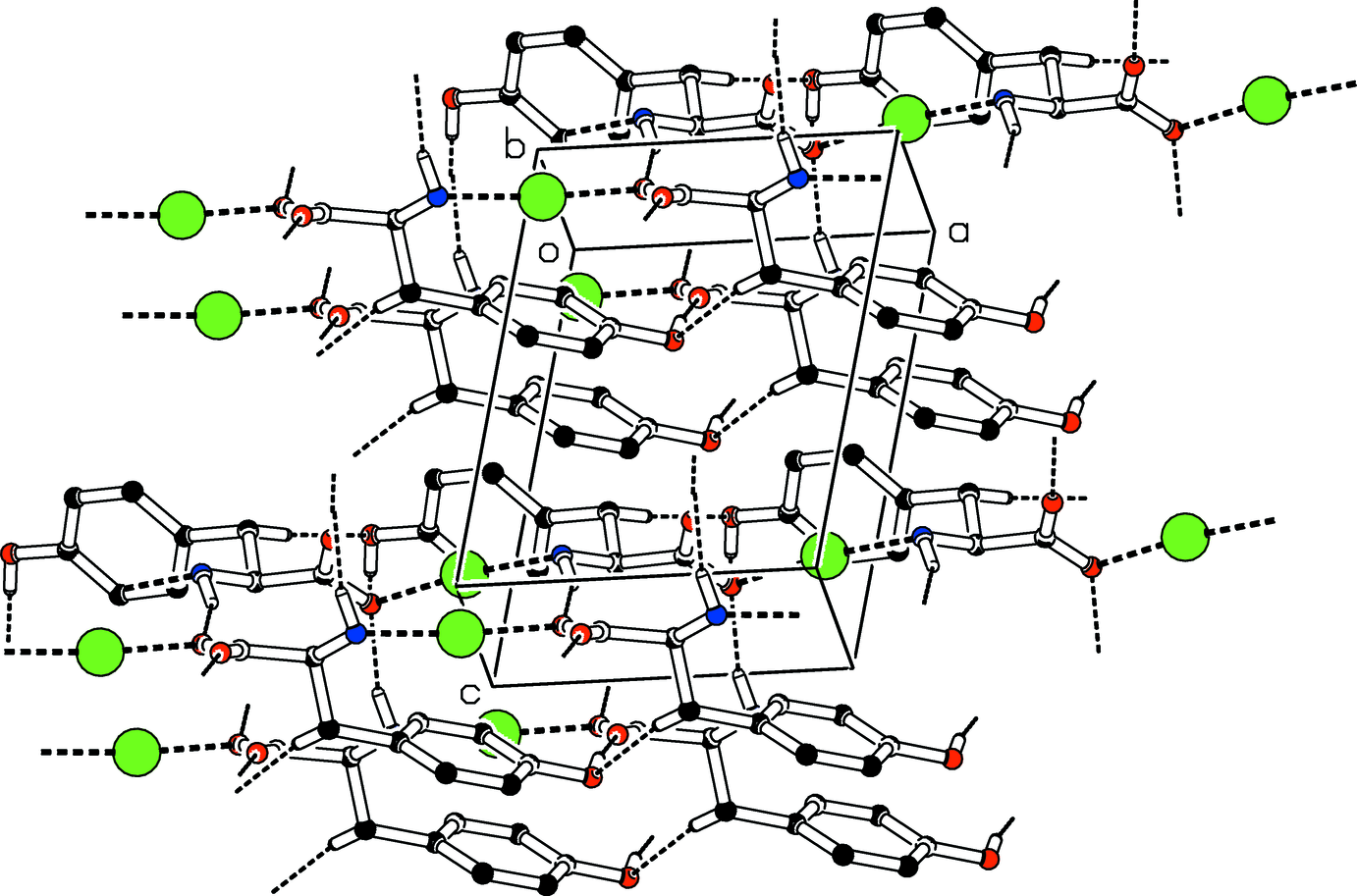

Supplement: Supplementary file 4 [file e-71-00m50-fig2.tif]
